# The Height Gain in Scoliotic Deformity Correction: Assessed by New Predictive Formula

**DOI:** 10.1155/2012/167021

**Published:** 2012-05-15

**Authors:** Ahmet Yılmaz Şarlak, Halil Atmaca, Resul Musaoğlu, Elşen Veli Veliev

**Affiliations:** ^1^Department of Orthopaedics and Traumatology, School of Medicine, Kocaeli University, Umuttepe Central Campus, 41380 Umuttepe, Kocaeli, Turkey; ^2^Department of Orthopaedics and Traumatology, Midyat State Hospital, Mardin, Turkey; ^3^Physics Department, Art and Science Faculty, Kocaeli University, Kocaeli, Turkey

## Abstract

Height gain after scoliosis correction is of a special interest for the patient and family. Ylikoski was the first to suggest a formula predicting height loss in untreated scoliotic patients. Stokes has recently suggested a new formula by using Cobb angle to determine height loss in idiopathic curves. We hypothesized that new additional variables to Cobb angle such as apical vertebral translation (AVT), number of instrumented segments (*N*), and disc heights may increase the accuracy of predicted height gain. According to our findings simple expression for height gain by simplified version of the formula is: *SP*Δ*H* = 0.0059*X*_1_ *θ*_1_ + 2.3(1 − (*θ*_2_/*θ*_1_))*N*, where *θ*_1_ is preoperative Cobb angle, *X*_1_ is preoperative AVT, *θ*_2_ is postoperative Cobb angle, and *N* is the number of instrumented vertebra. The purpose of this study is to analyze a new mathematical formula to predict height gain after scoliotic deformity correction.

## 1. Introduction

 A spine of a given length will have a lesser vertical height when curved, hence scoliosis is associated with height loss [1]. With the increasing importance of cosmetic concerns, short stature might be an important element of body dissatisfaction especially in adolescent patients with scoliotic deformity. There have been previous formulas curvilinearly correlating trunk height loss to the angulation of the primary curve. None of these formulas, however, had been confirmed clinically with respect to height gain in surgically corrected curves to our knowledge [1–4].

The purpose of this study is to analyze a new formula to predict height gain after scoliotic deformity correction taking into account the contribution of new variables (apical vertebral translation (AVT), number of instrumented segments (*N*), and disc heights) to increase accuracy which has not been used on previous formulas.

## 2. Materials and Methods

Thirty-six patients (30 females and 6 males) with single idiopathic curve treated by posterior instrumentation and fusion were analyzed. The mean age of the patients was 16.7 years (range. 7–45). The curves were classified according to Lenke classification system. Nineteen were type 1A, three type 1B, five type 1C, and nine type 5C. The heights of the patients were measured preoperative one week after surgery by the same resident who is unrelated to this study, and the difference between preoperative and postoperative body height noted as Δ*H* (mm). Standing long-cassette (36 × 14 inch) anteroposterior (AP) radiographs taken from a distance of 2 meters by the same X-ray machine (Toshiba Model: BLR-1000A) which was calibrated as each small square on radiograph that is equal to 1 cm in the world, at preoperative and immediate postoperative (3–7 days) were evaluated to determine the changes of Cobb angle (*θ*), AVT, and the number of the vertebra in the curve to predict the difference of body height (*P*Δ*H*) by using a new formula and the simplified version of this formula (*SP*Δ*H*). The radiographs were scanned in a computer workstation by using a transparent media scanner (Mikrotek MRS-3200A3). Digital software (Canvas 9.0) was used for analyzing the measurements. To determine the pre- and post-operative AVT, the centroid of the apical vertebrae was located at the intersection of lines connecting the superior lateral corners of the vertebral body to the contralateral inferior lateral corners. The horizontal distance from this point to the C7 plumb line (a line dropped parallel to the film edge from the like-determined centroid of C7) was the AVT for the proximal and main thoracic curves. For thoracolumbar/lumbar curves, the horizontal distance was measured from the center sacral vertical line (CSVL) (i.e. a line drawn vertically from the midline of the sacrum) [5]. The Δ*H*, *P*Δ*H*, *SP*Δ*H*, and the height loss by using the formula of Stokes [1] (Height loss (mm) = 1.0 + 0.066 ∗ Cobb + 0.0084 ∗ Cobb ∗ Cobb) was noted using Microsoft Office Excel 2003. Statistical analysis was performed using SPSS (version13.0; Chicago, USA). One-way ANOVA and post-hoc test (Tukey HSD) was used to compare the parameters and Kruskal-Wallis test used to find the correlation with the prediction of height gain and type of scoliosis. The differences between real height gain (Δ*H*) and three predictive formulas (*P*Δ*H*, *SP*Δ*H*, and Stokes^1^) were noted as Δ*H* − *P*Δ*H*, Δ*H* − *SP*Δ*H* and Δ*H*-Stokes^1^ for statistic analysis. All surgeries were performed by the senior author. The average number of instrumented vertebrae was 9.67 (range 4–14) (Table 1).

In critically analyzing the data in Table 1, the values that obtained by using Stokes^1^, formula differed from measured height gain (Δ*H*) in the majority of all cases after deformity correction. However. Stokes^1^ addressed only presumed height loss associated with a curve, without clinical validation. As also scoliosis is a three-dimensional deformity; correction maneuver that used during the surgery may change the distance between two vertebral bodies. To calculate the positive effect of intervertebral disc distance for each vertebra to height gain after correction; eleven patients (nine females and two males) were chosen from the study group that have ≤6.5°Cobb angle postoperatively (Table 2). The *k* value was calculated by using the formula below:

(1) $$k=\;\frac{\text{mean}\;\text{value}\;\text{of}\left(\Delta H\text{-}{\text{Stokes}}^{1}\right)}{N}.$$

### 2.1. Evaluation of the Formula

**Figure 1:**
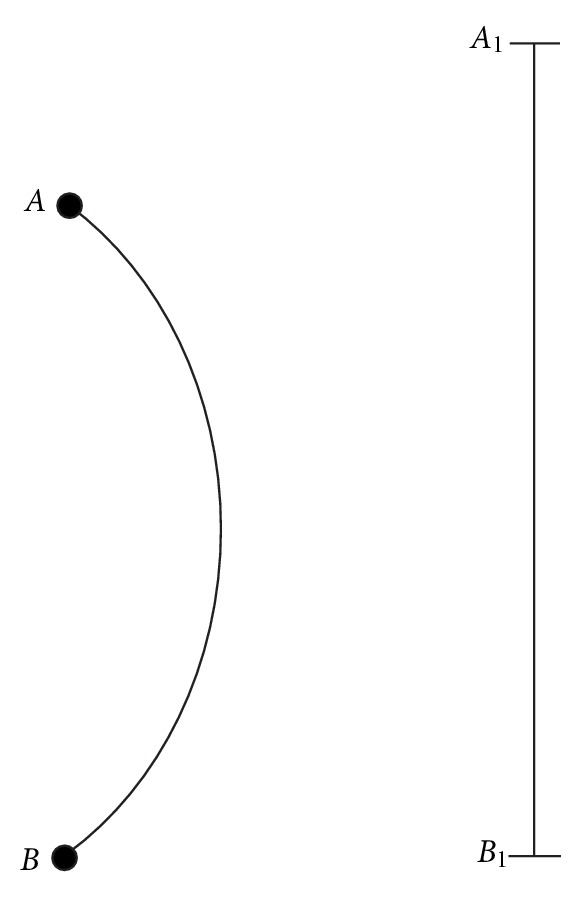
|*AB*| is the part of the preoperative spinal length. |*A*_1_ *B*_1_| is the postoperative view of the same segment.

When viewed in the coronal plane. the part of the preoperative curves is seen as in the form of *AB*, while we can measure the same segment as *A*_1_ *B*_1_  after surgery (Figure 1).

**Figure 2:**
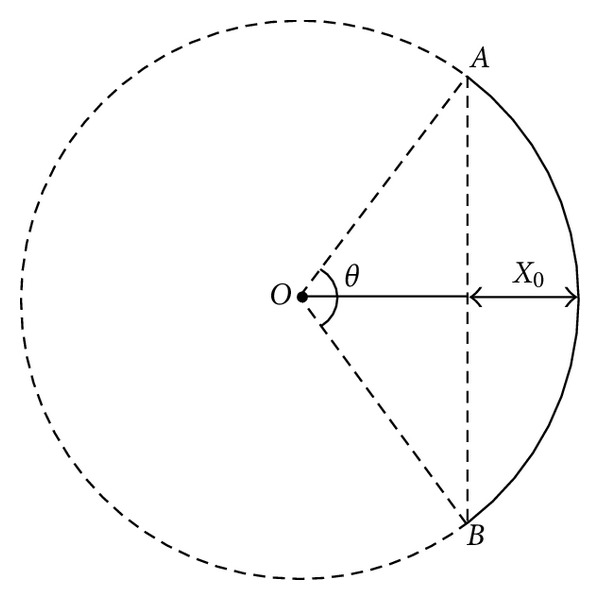
“*AB*” curve is the arc of a circle with center “*O*,” and the radius of this circle is *R*, the corresponding angle to the arc is *θ* (Cobb angle), while the AVT is *X*_0_.

Supposing that *AB* curve is the arc of a circle with center *O* and the radius of this circle is *R*, the corresponding angle to the arc is  *θ*, namely, Cobb angle (Figure 2). Cobb angle might be obtained by making a measurement on the plain radiographs of the patient.

The term for AVT was taken as *X*_0_ in the observed patients. From the triangular and circular geometry, the chord of *AB* and the arc-length of *AB* can be written as

(2) $$\begin{array}{c} \text{the}\;\text{chord}\;\text{length}\;\text{of}\;AB=2R\sin\left(\frac{\theta }{2}\right),\\ \text{the}\;\text{arc-length}\;\text{of}\;AB=\frac{2\pi R}{360}\theta . \end{array}$$

Here, *π* = 3.14 is the constant number. Providing that 100% correction is achieved after deformity correction, the geometrical contribution to the increase of height can be written as

(3) $$\begin{array}{cc} & \text{the}\;\text{arc-length}\;\text{of}\;AB-\text{the}\;\text{chord}\;\text{length}\;\text{of}\;AB\\ & \;=\frac{2\pi R}{360}\theta -2R\sin\left(\frac{\theta }{2}\right). \end{array}$$

On the other hand, due to taking the curved section as arc of circle, we can write the AVT as  *X*_0_ = *R*(1 − cos⁡(*θ*/2)). Using the formula the radius *R* of circle can be expressed in terms of AVT; *X*_0_  and of Cobb angle is *θ*:

(4) $$R=\frac{X_{0}}{1-\cos\left(\theta /2\right)}.$$

Consequently, the increase of height in a patient with 100% correction can be written as follows:

(5) $$\begin{array}{cc} & \text{the}\;\text{arc-length}\;\text{of}\;AB-\text{the}\;\text{chord}\;\text{length}\;\text{of}\;AB\\ & \;=\frac{2X_{0}}{1-\cos\left(\theta /2\right)}\left(\frac{\pi }{360}\theta -\sin\left(\frac{\theta }{2}\right)\right). \end{array}$$

The above formula has been obtained by assuming the spinal curvature forming an arc, considering the curvature as two-dimensional; although, the spinal curvature in scoliosis is three-dimensional. Therefore, the above formula might be an approximate formula considering only the Cobb angle.

 If we take into account contributions from intervertebral disc distance, *P*Δ*H* ^1^ might be used:

(6) $$P\Delta H¹=\frac{2X_{0}}{1-\cos\left(\theta /2\right)}\left(\frac{\;\pi \;}{360}\theta -\sin\left(\frac{\theta }{2}\right)\right)+aN.$$

Here, *a* is a constant parameter that was obtained from the 20 patients all have scoliotic curve. All patients were operated in our clinic and were not included in the current study. The preoperative and postoperative body heights of these patients were recorded, and the *a*  constant was found retrospectively by regression equation using the formula of *P*Δ*H* ^1^ to be 2.3 mm. The comparison of obtained *P*Δ*H*, *SP*Δ*H*, and Δ*H* is given in Table 1. As it is frequently impossible to get 100% correction in Cobb angle and AVT; we recalculated the *P*Δ*H* ^1^ for the patients that have residual deformities:

(7) $$\begin{array}{c} P\Delta H=h1-h+2.3\left(1-\frac{\theta_{2}}{\theta_{1}}\right)N, \end{array}$$

where

(8) $$\begin{array}{c} h1=\frac{2X_{1}}{1-\cos\left(\theta_{1}/2\right)}\left(\frac{\;\pi \;}{360}\theta_{1}-\sin\left(\frac{\theta_{1}}{2}\right)\right),\\ h2=\frac{2X_{2}}{1-\cos\left(\theta_{2}/2\right)}\left(\frac{\;\pi \;}{360}\theta_{2}-\sin\left(\frac{\theta_{2}}{2}\right)\right). \end{array}$$

And the final form of the formula is;

(9) $$\begin{array}{cc} P\Delta H & =\frac{2X_{1}}{1-\cos\left(\theta_{1}/2\right)}\left(\frac{\;\pi \;}{360}\theta_{1}-\sin\left(\frac{\theta_{1}}{2}\right)\right)\\ & \;-\frac{2X_{2}}{1-\cos\left(\theta_{2}/2\right)}\left(\frac{\;\pi \;}{360}\theta_{2}-\sin\left(\frac{\theta_{2}}{2}\right)\right)\\ & \;+2.3\left(1-\frac{\theta_{2}}{\theta_{1}}\right)N. \end{array}$$

In our predictive formula, *θ*_1_ is preoperative Cobb angle. *X*_1_ is preoperative AVT while  *θ*_2_ and  *X*_2_  is postoperative Cobb angle and AVT, *N*  is the number of vertebra that was instrumented.

 Although this formula contains all variables which may contribute to height gain, it is too complex and unusable in daily practice. In order to use this formula for practical calculation, we rewrite to simplify this expression using Taylor series method. The Taylor series of a function *f*(*x*) that is infinitely differentiable in a neighborhood of a real number *b* is the power series. As usually Cobb angles in scoliosis patients varies approximately between 35° and 75° we applied Taylor expansion to above formula as neighborhood of Cobb angle of 45°. As seen, our formula includes trigonometric functions sin⁡*θ* and  cos⁡*θ*. For example, Taylor series of sin⁡*θ* is as follows:

(10) $$\begin{array}{cc} \sin\theta & =\sin\theta_{0}+\left(\theta -\theta_{0}\right)\cos\theta_{0}\\ & \;-\frac{1}{2}{\left(\theta -\theta_{0}\right)}^{2}\sin\theta_{0}-\frac{1}{6}{\left(\theta -\theta_{0}\right)}^{3}\cos\theta_{0}. \end{array}$$

First, a few terms of this series are sufficient in our calculations. Using above expansion of sin⁡*θ* and similar expression for cos⁡*θ* after some simplifications, we obtain very simple expression for height gain:

(11) $$SP\Delta H=0.0059X_{1}\theta_{1}+2.3\left(1-\frac{\theta_{2}}{\theta_{1}}\right)N,$$

*θ*_1_: preoperative Cobb angle, *X*_1_: preoperative AVT, *θ*_2_: postoperative Cobb angle, *N*: the number of instrumented vertebra.

## 3. Results

The mean preoperative Cobb angle of 47.6° (range, 37.6°–75.2°) was corrected to 12.4° (range, 0°–41.2°). The mean preoperative and postoperative AVT was 44.4 mm (range, 10.1 mm–86.5 mm) and 11.0 mm (range, 0 mm–48.3 mm), respectively; the mean Δ*H* was 28.53 mm (range, 15 mm–50 mm). The mean *P*Δ*H* that measured by using new formula was 28.26 mm (range, 12.46 mm–45.9 mm). The mean *SP*Δ*H* was 29.57 mm (range, 14.56 mm–51.89 mm). The mean value of the height gain that was noted using the Stokes^1^ formula was 20.26 mm (range, 12.52 mm–48.88 mm). The mean Δ*H*-*P*Δ*H* was 0.27 mm (standard deviation = 9.26), and the mean Δ*H*-*SP*Δ*H* was −1.04 mm (standard deviation = 9.51), while Δ*H*-Stokes^1^ was 8.27 mm (standard deviation = 9.77). Although there were significant differences between Δ*H*-Stokes^1^ with either Δ*H*-*P*Δ*H* (*P* = 0.002) or Δ*H*-*SP*Δ*H* (*P* = 0.000), there was no significant difference between Δ*H*-*P*Δ*H* and Δ*H*-*SP*Δ*H* (*P* = 0.829). There was no significant correlation between curve type and three predictive formulas (*P* = 0.265 for Δ*H*-*P*Δ*H*. *P* = 0.279 for Δ*H*-*SP*Δ*H*, and *P* = 0.561 for Δ*H*-Stokes^1^). The mean *k* value was (2 ± 0.70) mm (Table 2).

## 4. Discussion

In the early conservative treatment periods of 1950–1960s, the height gain measurement in scoliosis patients was aimed to assess vital capacity [2.6.7]. Archer and Dickson mentioned about spinal height gain due to kyphoscoliosis correction in 1985 [6]. Ylikoski was the first to suggest a formula predicting height loss in untreated scoliotic patients [4]. Stokes has recently suggested a new formula to determine height loss in idiopathic curves [1]. None of these previous formulas on height loss attributed to the deformity, however, has had clinical correlation. 

**Figure 3:**
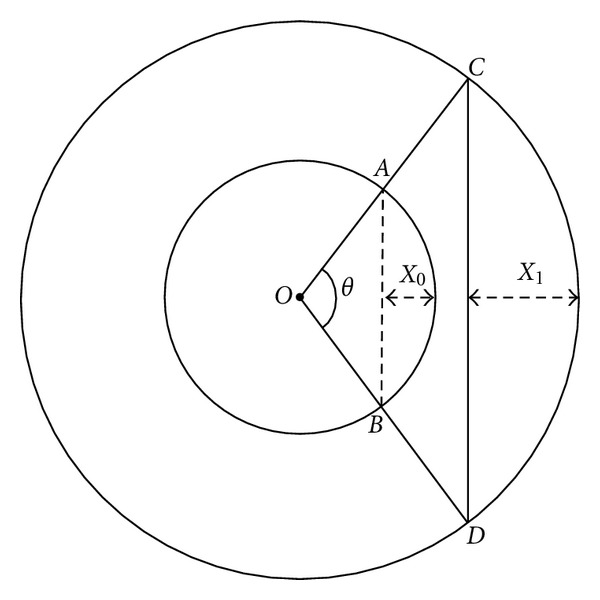
|*AB* | ≠|*CD*| and *X*_0_ ≠ *X*_1_, although these two arcs have the same Cobb angle (*θ*).

Our hypothesis like that of Brookenthal [3] assumes a circular geometry of the scoliotic curve. Stokes assumed that spinal height occurs only as a result of altered curvature without alteration in disc height [1]. While the relationship between height loss and Cobb angle was determined by regression analyses, it is obvious that for the same Cobb angle the spinal length and height may change with respect to AVT and number of instrumented vertebrae that has not been taken into consideration by Stokes [1] (Figure 3). 

Scoliotic curvature of the spine results from a combination of disc and vertebral wedging. Our analyses in clinical setting showed Stokes' formula to underestimate height gain after deformity correction. To find out contribution of each disc space to height gain, we have used the formula to find “*k*” value: *k* = mean value of (Δ*H*-Stokes^1^)/*N*. The “*k*” value being equal to “*a*” constant in our regression analyses shows the reliability of the “*k*” value. This suggests that the surgery increased disc height while reducing the Cobb angle. 

 Stokes quantified the spinal deformity by the average of two Cobb angles in double curves whether or not both were considered structural [1]. Our analysis does not compromise sufficient number of patients having double curves to comment on height gain in such patients. Mean errors ranging from 1.7° to 6.5° in both manual and digital measure of Cobb angle [7–11], two-dimensional measurement of a three-dimensional deformity, possible erroneous height measurements in clinical setting are the main limitations of this study. However, our application of the formula to scoliosis patients suggests being applicable. 

 In idiopathic scoliosis, the convex sides of bodies. Discs, pedicles, intervertebral foramina, and lamina are greater than the depths of the same structures on the concave side [12]. During posterior instrumented deformity correction by rod derotation, translation maneuver of posterior elements on the concave side is lengthened. Thus, the concave-sided rod gets shorter than the measured curve length. The present study was motivated by the need to estimate percent curve correction intraoperatively by the relative rod shortening on the concave side using our new simplified predictive mathematical formula:

(12) $$SP\Delta H=0.0059X_{1}\theta_{1}+2.3\left(1-\frac{\theta_{2}}{\theta_{1}}\right)N.$$

To illustrate the results, an individual patient is presented with a single thoracic curve of 50° Cobb angle and 30 mm AVT. We have planned eight-vertebra instrumentation and 70% correction with derotation maneuver. Therefore, final Cobb angle must be 15° postoperatively. Using our simplified formula:

(13) $$SP\Delta H=0.0059*30*50+2.3\left(1-\frac{15}{50}\right)8.$$

*SP*Δ*H* = 21.73 mm; likewise, we have to cut our concave sided rod 21.73 mm longer than the measured spinal height intraoperatively to achieve the planned 70% correction. It is interesting to note that for two right thoracic scoliotic deformities with equal Cobb angle spinal height may change significantly with even a single change in the number instrumented vertebra. Estimating percent curve correction intraoperatively by rod length, however, is hindered by many factors which if solved may in future serve as a tool to optimize instrumentation. If we quantitate curve correction, it may also help to prevent trunk imbalance in the frontal plane that is sometimes encountered with posterior derotational systems. Using the formulas from the measurements in the intraoperative X-rays may solve many of the problems due to patient positioning, anesthesia reducing spine stiffness, and calibration problems causing sizable changes in the exact measure of spinal height by preoperative radiographs. 

 Definition of the scoliotic deformity as an arc is an oversimplification of the three-dimensional deformity. Relying on two-dimensional measurement as well as not considering the compensatory curves are the main shortcomings of our study. Also the current formula may be more appreciable to younger patients with relatively small curves without vertebral body deformation.

## 5. Conclusion

From the findings in this study, it would appear that AVT. number of instrumented vertebra and “*k*” value of (2 ± 0.70) mm for each instrumented disc space offers a significant additional parameter to Cobb angle to achieve an almost perfect prediction of postoperative height gain in idiopathic scoliosis.

## Figures and Tables

**Table 1:** Variable and measured data of the patients.

Number	Age	Gender	Curve Type	Fusion level	*N*	Preop. Cobb (°)	Preop. AVT (mm)	*h*1 (mm)	Postop. Cobb (°)	Postop AVT (mm)	*h*2 (mm)	H1 (mm)	*PΔH* (mm)	Simplified *P*Δ*H* (mm)	Stokes^1^ (mm)	Δ*H* (mm)	Δ*H*-*P*Δ*H* (mm)	Δ*H*-*SP*Δ*H* (mm)	Δ*H*-Stokes^1^ (mm)
(1)	16	F	1B	T4-L4	13	51.2	46.5	43.61	22.8	17.2	2.10	41.51	28.21	30.63	22.73	30	1.79	−0.63	7.27
(2)	14	F	1A	T6–T11	6	45.9	50.0	26.97	16.3	19.6	1.58	25.39	20.49	22.44	18.35	40	19.51	17.56	21.65
(3)	14	F	1A	T5-L1	9	45.4	37.2	30.39	14.7	10.9	0.76	29.63	22.92	23.96	17.98	30	7.08	6.04	12.02
(4)	45	F	1A	T4-L3	12	56.5	54.7	45.50	28.6	26.3	4.17	41.33	27.35	31.86	27.60	15	−12.35	−16.86	−12.60
(5)	14	M	1A	T3-L1	11	43.7	51.4	38.15	6.5	10.1	0.02	38.13	34.37	34.79	16.68	30	−4.37	−4.79	13.32
(6)	17	F	5C	T11-L5	7	45.5	21.2	21.63	13.5	2.0	0.12	21.51	16.72	17.01	18.04	18	1.28	0.99	−0.04
(7)	14	F	1A	T5-L1	9	41.2	49.4	32.33	16.1	23.5	1.86	30.47	22.39	24.65	14.90	20	−2.39	−4.65	5.10
(8)	13	F	1A	T5–T11	7	38.4	32.5	23.19	13.7	20.5	1.29	21.90	16.15	17.71	13.01	15	−1.15	−2.71	1.99
(9)	13	F	5C	T11-L4	6	37.6	49.6	24.39	0.6	8.8	−3.38	27.77	27.55	24.59	12.52	30	2.45	5.41	17.48
(10)	17	M	1A	T2-L1	12	41.3	55.0	40.57	7.0	22.1	0.17	40.39	35.70	36.31	14.96	20	−15.70	−16.31	5.04
(11)	13	F	5C	T10-L4	7	42.5	76.9	34.79	7.6	27.7	0.39	34.41	31.52	32.51	15.83	30	−1.52	−2.51	14.17
(12)	14	F	5C	T3-L4	14	48.5	47.5	45.46	13.1	15.0	0.88	44.58	35.87	37.09	20.44	38	2.13	0.91	17.56
(13)	20	F	1A	T4–T11	8	38.0	38.8	26.77	5.4	5.9	−0.07	26.84	24.22	24.48	12.74	25	0.78	0.52	12.26
(14)	18	M	5C	T6-L4	11	48.7	26.5	32.74	17.8	5.2	0.47	32.27	23.03	23.69	20.63	30	6.97	6.31	9.37
(15)	37	F	1C	T4-L1	10	52.4	27.5	31.32	10.8	4.3	0.18	31.14	26.42	26.78	23.82	27	0.58	0.22	3.18
(16)	12	F	1C	T2-L3	14	64.4	33.2	44.64	10.3	0.0	0.00	44.64	39.52	39.68	35.84	30	−9.52	−9.68	−5.84
(17)	7	F	1A	T4-L2	11	39.6	32.1	32.53	10.1	0.0	0.00	32.53	26.08	26.34	13.80	20	−6.08	−6.34	6.20
(18)	13	F	1A	T5–T11	7	39.0	26.0	21.86	10.0	0.0	0.00	21.86	17.72	17.94	13.37	22	4.28	4.06	8.63
(19)	18	F	1B	T6-L4	11	45.3	53.0	39.06	6.2	0.0	0.00	39.06	35.59	35.99	17.87	35	−0.59	−0.99	17.13
(20)	14	F	1A	T5–T12	8	46.2	53.0	32.46	15.4	9.5	0.71	31.75	25.62	26.72	18.59	25	−0.62	−1.72	6.41
(21)	14	F	1A	T3-L3	13	75.2	86.5	68.01	41.2	48.3	11.34	56.66	40.29	51.89	48.88	40	−0.29	−11.89	−8.88
(22)	21	F	1C	T4–T11	8	42.8	24.0	24.27	3.7	1.1	0.00	24.27	22.68	22.87	16.02	40	17.32	17.13	23.98
(23)	14	F	1B	T5–T11	7	39.6	38.0	24.66	6.5	0.0	0.00	24.66	22.02	22.33	13.78	30	7.98	7.67	16.22
(24)	20	F	1C	T4-L3	12	46.2	10.1	30.29	5.0	0.0	0.00	30.29	27.30	27.38	18.61	35	7.70	7.62	16.39
(25)	14	F	1A	T7-L2	8	50.0	58.6	35.26	15.4	17.9	1.33	33.94	28.27	30.01	21.65	30	1.73	−0.01	8.35
(26)	14	M	1C	T2-L3	14	63.5	84.1	63.27	27.9	31.5	4.85	58.42	44.30	49.58	34.83	50	5.70	0.42	15.17
(27)	16	M	5C	T11-L2	4	45.5	34.6	18.24	19.5	18.2	1.84	16.39	12.46	14.56	18.05	15	2.54	0.44	−3.05
(28)	15	F	5C	T5-L5	13	52.1	53.2	45.90	0.0	0.0	0.00	45.90	45.90	46.26	23.56	35	−10.90	−11.26	11.44
(29)	12	F	1A	T4–T12	9	50.5	47.5	34.52	14.3	0.0	0.00	34.52	28.68	29.00	22.11	30	1.32	1.00	7.89
(30)	12	M	1A	T4–T11	8	42.7	20.3	23.36	5.0	0.0	0.00	23.36	21.21	21.37	15.98	35	13.79	13.63	19.02
(31)	12	F	5C	L1–L4	4	53.4	48.1	24.01	31.4	24.3	4.26	19.75	14.35	18.92	24.65	23	8.65	4.08	−1.65
(32)	25	F	1A	T4-L3	12	53.9	58.3	45.77	7.9	6.3	0.10	45.67	41.65	42.13	25.19	15	−26.65	−27.13	−10.19
(33)	12	F	1A	T3-L2	11	49.1	35.3	35.27	12.3	10.6	0.56	34.71	28.37	29.18	20.91	15	−13.37	−14.18	−5.91
(34)	20	F	1A	T4-L4	13	46.9	43.8	41.69	10.7	8.7	0.35	41.34	34.55	35.22	19.11	24	−10.55	−11.22	4.89
(35)	14	F	5C	T9-L4	8	50.3	45.9	31.69	0.0	0.0	0.00	31.69	31.69	32.01	21.91	40	8.31	7.99	18.09
(36)	24	F	1A	T5-L3	11	40.3	47.1	36.11	0.0	0.0	0.00	36.11	36.11	36.49	14.25	40	3.89	3.51	25.75

**Mean**	**16.7**				**9.67**	**47.6**	**44.4**	**34.74**	**12.4**	**11.0**	**1.00**	**33.74**	**28.26**	**29.57**	**20.26**	**28.53**	**0.27**	**−1.04**	**8.27**

**Table 2:** Variable and measured data of the patients who were chosen from the study group to find “*k*” value.

Number	Age	Gender	Curve type	Fusion level	*N*	Preop. Cobb (°)	Preop. AVT (mm)	*h*1 (mm)	Postop. Cobb (°)	Postop. AVT (mm)	*h*2 (mm)	H1 (mm)	*P*Δ*H* (mm)	Stokes^1^ (mm)	Simplified *P*Δ*H* (mm)	Δ*H* (mm)	Δ*H*-Stokes^1^ (mm)	Intervert. disc lengthening (“*k*” value)
(5)	14	M	1A	T3–L1	11	43.7	51.4	38.15	6.5	10.1	0.02	38.13	34.37	16.68	34.79	30	13.32	1.21
(9)	13	F	5C	T11–L4	6	37.6	49.6	24.39	0.6	8.8	−3.38	27.77	27.55	12.52	24.59	30	17.48	2.91
(13)	20	F	1A	T4–T11	8	38.0	38.8	26.77	5.4	5.9	−0.07	26.84	24.22	12.74	24.48	25	12.26	1.53
(19)	18	F	1B	T6-L4	11	45.3	53.0	39.06	6.2	0.0	0.00	39.06	35.59	17.87	35.99	35	17.13	1.56
(22)	21	F	1C	T4–T11	8	42.8	24.0	24.27	3.7	1.1	0.00	24.27	22.68	16.02	22.87	40	23.98	3.00
(23)	14	F	1B	T5–T11	7	39.6	38.0	24.66	6.5	0.0	0.00	24.66	22.02	13.78	22.33	30	16.22	2.32
(24)	20	F	1C	T4-L3	12	46.2	10.1	30.29	5.0	0.0	0.00	30.29	27.30	18.61	27.38	35	16.39	1.37
(28)	15	F	5C	T5-L5	13	52.1	53.2	45.90	0.0	0.0	0.00	45.90	45.90	23.56	46.26	35	11.44	0.88
(30)	12	M	1A	T4–T11	8	42.7	20.3	23.36	5.0	0.0	0.00	23.36	21.21	15.98	21.37	35	19.02	2.38
(35)	14	F	5C	T9-L4	8	50.3	45.9	31.69	0.0	0.0	0.00	31.69	31.69	21.91	32.01	40	18.09	2.26
(36)	24	F	1A	T5-L3	11	40.3	47.1	36.11	0.0	0.0	0.00	36.11	36.11	14.25	36.49	40	25.75	2.34

**Mean**	**16.8**				**9.4**	**43.5**	**39.2**	**31.33**	**3.5**	**2.4**	**−0.31**	**31.64**	**29.88**	**16.72**	**29.87**	**34.09**	**17.37**	**2.0**
